# Reports on the Hospitals of the United Kingdom

**Published:** 1909-11-13

**Authors:** Henry Burdett


### REPORTS
ON THE
### Hospitals of the United Kingdom.

By SIR HENRY BURDETT, K.C.B., K.C.V.O.

### VIII.—THE SALISBURY INFIRMARY.

This institution was founded 143 years ago. During the whole period of its existence it has faithfully discharged the heavy responsibility of providing adequate treatment and care for the sick and suffering poor of Salisbury and the district for many miles round. It is seventeen years since we last inspected this institution, and it happened that we met on the staircase during our visit Mr. H. L. Wilks, now one of the surgeons, who was house surgeon in 1892. We compared notes, and both agreed that the present condition of every department of the Salisbury Infirmary presented features of marked improvement compared with what they were seventeen years ago. It is probable, great as the changes for good in many directions have been, that the best asset Salisbury Infirmary has to-day is the spirit which animates all the staff, from the highest to the lowest; and this excellent spirit makes itself felt throughout the whole establishment. It dominates and penetrates everything.

### An Ideal for Residents.

Forty years ago, twenty years ago, and even more recently, many county hospitals were but infirmaries where many of the beds were frequently occupied by chronic cases. To-day the Salisbury Infirmary is conducted—as all county hospitals should be—on identical lines with an up-to-date clinical hospital. This fact, coupled with the knowledge that the accommodation provided for the resident medical staff is both attractive and comfortable, that excellent sport, fishing, shooting, golf, and so forth are easily obtainable, makes these resident appointments at Salisbury almost ideal for a recently qualified man who is ambitious to perfect his practical knowledge of his profession and to live a happy life.

### The Present Buildings.

Architects and all hospital managers who desire to perfect themselves in practical knowledge, and to learn what is material in hospital construction where a due regard to economical considerations is maintained, may learn much by a visit of inspection to this institution. Many of the wards are antiquated and long out of date. Some of them, the better ones, represent merely the middle period between structural imperfection and a gradual improvement in hospital buildings based on hygienic principles. Yet these buildings as they exist to-day exhibit few remediable defects, and the enforcement of the strictest attention to cleanliness and order to an extent which has proved them to be safe and even pleasant sick-rooms for the treatment of patients suffering from surgical ailments of all kinds. The experience of Salisbury proves that the aseptic treatment, combined with the best nursing and the highest administration and efficiency, renders the mere building in which disease is now treated a matter of relative unimportance. All honour to the staff, resident and honorary, who have brought the Salisbury Infirmary to its present high state of efficiency.

### Finance.

Like most county infirmaries, Salisbury needs more cash. The outstanding balance last year, with the amount brought forward, made the sum owing to the Treasurer upwards of £2,000. The cause of this deficiency is mainly due to the existing ticket system, whereby a subscriber of two guineas receives back in value, as represented by patients treated, quite 200 per cent. of the money he hands in to the Treasurer each year. This is not charity at all, but patronage of the worst type, for it is pretentious humbug to talk of charity in connection with a system which enables any one person to pose as a philanthropist at the expense of a previous generation and of some of his more conscientious and liberal neighbours. Salisbury is not quite so bad in this respect as some institutions, but the whole of the privileges of subscribers and governors need overhauling. If this is wisely done by a special committee of the governors the hospital should soon be in funds, and its income should prove adequate to meet its expenditure for many years to come.

### Wanted, a Private Nursing Staff.

Another point which all county hospitals which depend on the voluntary system for their income must keep in mind is that the present trend of legislation and of parliamentary action is hostile to sympathy between one class and another. The governors of county hospitals which have to provide for the needs of the sick poor in their midst must lose no opportunity to identify their work with the requirements of the community which their institution immediately serves. All classes want the services of trained nurses in the day of sickness, and a county hospital should maintain a sufficiently large private staff of nurses to enable all its subscribers to be guaranteed the services of an efficient nurse whenever required. The profits made on the private nursing staff should permit an adequate system of payment, pension and sick pay provision for all the nurses to be set up, and place a sum at the disposal of the authorities which would enable many necessary things to be done which could not otherwise be undertaken. In this way the whole of the community, realising as they must how indispensably necessary the county hospital is to their protection and comfort, will continue to provide in increasing quantity all the money found necessary from time to time for its efficient maintenance.

### IX.—SOME COTTAGE HOSPITALS.

### ASHBURTON AND BUCKFASTLEIGH COTTAGE HOSPITAL.

This hospital was established in 1876 and rebuilt in 1889. The new building is of stone, placed upon an elevated site, with an attractive exterior, beautiful views, and pretty grounds. It contains 10 beds. The plan is very simple and easy to work. Though built so recently, the w.c.'s and slop sinks are out of date and should be replaced. The matron was away, and it was a great disappointment to find an absence of neatness and order throughout the hospital, especially as the lavatories and other arrangements were none too clean. This is the more surprising, for 16 abdominal operations, and 25 others under an anæsthetic, were performed here during the year. Seeing that the medical staff acknowledge their obligations to the nurses, and testify to their skill and devotion, the condition of the hospital as we found it in the matron's absence may, we hope, be put down to temporary causes.

### CHIPPENHAM COTTAGE HOSPITAL.

This hospital was not designed by an architect who had a thorough knowledge of hospital hygiene and requirements. The new men's ward, the new slop sinks and kitchen are a great improvement upon the original plan, though this is only 10 years old. This hospital illustrates, like that at Cirencester, the vital importance of selecting an able, energetic, and capable nurse, with good organising powers, for the post of matron. Miss E. Stevens, who was trained at Guy's Hospital, does credit to her school. She has proved an excellent matron, and has her hospital in perfect order throughout. There is here a place for everything, and everything is in its place. Miss Stevens was appointed matron 10 years ago, and she has done everything possible to minimise the defects of the original plan. The beds in the children's ward are too large and give it an overcrowded appearance. The mortuary, like that at most cottage hospitals, contains no adequate fittings or appliances for post-mortems, and is without any water supply. Yet, as we have insisted over and over again, the mortuary of every hospital, large or small, should never be used as a store or lumber room, and should always exhibit respect for the dead, and be kept clean and in the most perfect order. These defects ought to be remedied at once. In other respects the Chippenham Cottage Hospital is well managed and commendable.

### CIRENCESTER COTTAGE HOSPITAL.

This is one of the most interesting cottage hospitals in the world. It illustrates that Albert Napper's ideal of 1859 can be completely realised, for it proves that every sort of case, however complicated, whether medical or surgical, can be successfully treated in a cottage hospital. We congratulate the people of Cirencester and the district on the possession of so able and skilled a staff of practitioners as that attached to their cottage hospital. Like the inhabitants of Marlborough, which is blessed with the Savernake Cottage Hospital and its able medical staff, all classes of the population of Cirencester, both rich and poor, can secure the best medical and surgical skill and attendance in their own homes. These are results which the cottage hospital was instituted to bring about. They are everywhere capable of accomplishment, and where a cottage hospital fails in these respects it is a failure and has little justification for its continuance. The Cirencester Cottage Hospital has a most attractive elevation, with a large, open, roofed-in verandah leading to the front door. The whole hospital is quaint but well equipped. It contains everything essential to the adequate treatment of disease and the comfort of the patients. No case is ever sent off to a larger hospital because the staff cannot deal with it, as is the case at some cottage hospitals. Every cottage hospital committee should look into this question of thoroughly efficient medical and surgical service. If they do not possess it at present they should take steps to secure it without delay. The wards, cupboards, kitchens, fittings, mortuary, theatre, and larder are all beautifully kept, and are adequate and sufficient. Good sense dominates the management, and makes good order and smartness prevail throughout the hospital. In short, the Cirencester Cottage Hospital is up-to-date, does all kinds of medical and surgical work efficiently and well in a quiet, methodical, and effective way. The matron, Miss M. S. Warner, has held her present office for twelve years, to the great satisfaction of everybody, and to the advantage of the hospital and its patients.

### EVESHAM COTTAGE HOSPITAL.

This hospital was established in 1879, and since then several additions have been made to it, and a new operating theatre has been built. The chief wards were being extended at the time of our visit, and a verandah, which is too narrow, has been added to the top floor ward. The ground floor has no verandah, but merely a heavy concrete descending platform to enable patients to be wheeled out into the garden. It would be far better to have a concrete floor the whole length of the ground floor ward, and to have it and the upstairs verandah roofed in with glass, the width of both verandahs being sufficient to enable a bed to stand lengthways and at right angles to the outside wall of the ward. It is a well-kept, well-found, bright, and comfortable little hospital, the condition of which reflects the greatest credit on the matron, Miss M. Hipkins, who has everything in good order. Like most cottage hospitals, the mortuary, so-called, is not creditable to the management. We hope that the Committee will realise that it is time to make it worthy of the institution by putting it into thorough working order, with the necessary fittings, appliances, etc., and make it an object lesson to the community the hospital serves, by exhibiting a proper respect for the dead and a fitting sense of what is due to the living.

**The Sussex County Hospital, Brighton,** will be reported on next week.

